# Screening Scheme Evaluation of the Assembly Process Based on the Stress-Strength Model and Defect Stream Analysis

**DOI:** 10.3390/e20060447

**Published:** 2018-06-07

**Authors:** Yubing Huang, Wei Dai, Lianxi Liu, Yu Zhao

**Affiliations:** 1 School of Reliability and Systems Engineering, Beihang University, Beijing 100191, China; 2 Beijing Xinghang Mechanical-Electrical Equipment Co., Beijing 10091, China

**Keywords:** assembly process, screening evaluation, defect stream, stress-strength model, entropy

## Abstract

During the assembly process, there are inevitable variations and noise factors in the material properties, process parameters and screening scheme, which may affect the quality of the product. Using the stress-strength model, an evaluated screening scheme method, by analyzing the variation of the defect density in the assembly process, is proposed and discussed. The influence of screening stress on product defects is considered to determine the screening scheme. We performed the defect stream analysis by calculating the recursive relations of residual defect density under multi-stress conditions. We find that the probability density function, which shows the defect changing process from latent to dominant relative to the time process, agrees very well with the historical data. We also calculate the risk as the entropy of the assembly task. Finally, we verify our method by analyzing the assembly process of a certain product.

## 1. Introduction

In the lifecycle of mechanical product manufacturing, the quality of the product depends on the quality of the assembly process [1]. A fatal or killer defect is one that is of sufficient size and occurs in a place where the outcome is an immediate device failure [2], for example, when the product size exceeds the design threshold after processing; such a defect is detected after the manufacturing or screening test. A defect that is either too small or is located in a position that does not cause an immediate failure is called a latent defect. A latent defect may or may not cause failure in the field, depending on the operation time, environmental condition and processes [3].

In the evolution of the potential defects, potential defect analysis is performed to determine the dominant influence on the reliability of the product, which can help to determine the most reliable assembly sequence and screening position. Previously, there were some attempts to extend defect models to estimate reliability. A simple time-independent Poisson reliability was obtained by directly assuming that the number of nonfatal defects is proportional to the number of fatal defects following a Poisson distribution [4,5,6]. But only cases of the distribution of defects following a certain law are analyzed and they were not completely applied to the assembly process. In addition, all these models implicitly assumed that the number of fatal defects is independent of the number of the latent defects in a device.

Different resistance strengths of products corresponding to different stresses in the assembly process will result in different defects if the strength threshold is exceeded. Whitney et al. [7,8] presented a residual strength degradation model which deals with both tensile and compressive strength degradation as competing failure modes. Xie et al. [9] established a direct correlation between the failure rate and failure-controlling variables and analyzed the causal relationship between the failure rate curve shape and the probabilistic property of the load-strength interaction, developing a load-strength interference relationship-based failure rate model. Despite the extensive studies on competing failure models, we notice that few works have focused on the impact of stresses on different failure modes. This motivates us to investigate the impacts of different stresses from the perspective of the failure rate [10]. Such processes are often “competing”, leading to failure [11] and may be dependent on each other in a number of ways; in this case they are referred to as dependent competing faire processes [12]. Various dependencies have been discussed in the literature, e.g., the statistical dependency described by joint probability distributions [13,14], copulas [15,16], and the functional dependency described by failure propagation and isolation [17,18,19,20,21]. In this paper, we consider the dependency between two types of failure. Different screening methods aim at different product defects, so the first innovation of this paper is to establish the relationship between stress and defect by quantifying the stress of the assembly process, so as to optimize the selection parameters more precisely. Models consider both degradation and the relationship between stress and strength during the assembly process.

Environmental stress screening (ESS) refers to the process of exposing a newly manufactured or repaired product or component (typically electronic) to stresses such as thermal cycling and vibration in order to force latent defects to manifest themselves by permanent or catastrophic failure during the screening process. Although there have been some empirical studies on the ESS in the literature, the study conducted by Cha and Finkelstein et al. [22,23,24] is the first to deal with adequate stochastic modeling, analysis and optimization of the ESS. Elskamp and Peng et al. [25,26] analyzed the effect of the screening; Kumar and Omar [27] quantized the screening procedure; Khatibi and Suhir et al. [28,29,30] studied the electronic parts with the ESS. However, the screening process was not quantitatively analyzed from the perspective of defect. The second innovation of this paper is to optimize the screening parameters in the selection scheme by quantifying the defect density of the product in the assembly process.

The paper is organized as follows. In the next section, we use the stress-strength model to analyze defect generation in the assembly unit. Then, an assembly process model is developed to describe the defect stream during this process, which considers the defect changing process from latent to dominant relative to the time process. The failure efficiency of the product under different stress levels is calculated by considering the multi-stress strength interference model under the result of competitive failure. Then, the risk of the improved screening method is evaluated by calculating the change in the defect density. Finally, an example related to a printed circuit board assembly (PCBA) is analyzed by our model.

## 2. Stress-Strength Model in Assembly Unit

### 2.1. Defect Analysis in Assembly Unit 

The quality of assembly is the result of the interaction of multiple processes. Each process has the effect of optimizing the quality of the product and increasing the potential defects of the product. There is also the stimulation and correction of the potential defects of the product between the processes. In other words, each process is the correction and inheritance of the potential defects left over from the previous processes. Therefore, this section analyses the defect generation and rejection of the single process in the assembly.

**Figure 1:**
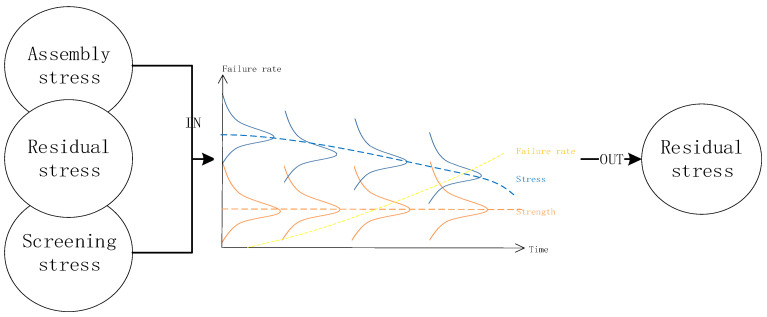
Defect analysis in assembly unit.

As shown in Figure 1, potential defects may be introduced in the assembly process or the potential defects may be fired as a dominant defect during the assembly task. The model developed in this paper is motivated by the actual engineering problem of modeling the analysis of printed circuit board (PCB) assembly. A PCB is subject to two main failure mechanisms: one concerns the electro- migration, the other one involves the component solderability. This paper assumes that the assembly phase of each process is subject to the same distribution D and the defect is limited for each process to a single stress action. Therefore, the stress strength interference model based on the competitive failure algorithm is established to analyze the resistance strength of PCB under different stresses, and the product failure efficiency under corresponding stress is obtained.

### 2.2. Stress-Strength Model with Competing Failure

For structural reliability, the failure of a system occurs when the stress modeled by an external shock process exceeds the strength of the system for the first time. In practice, the probability of this failure depends on the magnitudes of the previously survived shocks. The stress-strength interference model and reliability calculation formula are the basis of reliability design and failure analysis [31], but in many cases they are difficult to direct. The main reason for the application of engineering calculation is:

1. Lack of distribution data;
2. There are several random variables affecting product reliability, and the stress-strength interference model cannot be applied directly to n-dimensional vectors.

In this paper, according to the known requirements of product reliability and the stress distribution in the assembly process, because the assembly process is too complicated to express the strength completely with a distribution, the product strength requirement that satisfies the product reliability requirement under the stress of the assembly process is obtained by a linear search method. The product strength obtained is taken as the standard to measure whether the assembly process can meet the product quality. The residual defects affect the strength of the product, so the relationship between the potential defects, the stress in the assembly process, and the reliability of the product is established.

We have defined the auxiliary function $Z=G(x_{1},\;\ldots ,\;x_{n},\;x_{n+1},\;\ldots ,\;x_{2n})$, where *x_i_* (*i* = 1, 2, ..., *n*) represents various random stresses during the assembly process and *x_n+i_* is the strength under *x_i_* stress. This function is called a multivariate function, representing the state of the product. When *Z* > 0, the intensity is greater than the stress; when *Z* < 0, the strength is lower than the stress. Let *x_i_* follow a normal distribution with an average *μ_i_* and a standard deviation *σ_i_*; according to the linear search method, the main steps are as follows:

Step 1: Assign the initial value $\left(x*)=(x_{1}*,\cdots ,\;x_{n}*,\;x_{n+1}*,\;\cdots ,\;x_{2n}*\right)$ to each random variable, generally taking the average value of each variable;
Step 2: Compute the partial differentiation of function *Z* at the current value point of each random variable $\frac{\partial G}{\partial x_{i}}(i=1,2,\cdots ,2n)$;
Step 3: Compute the sensitivity coefficient $\zeta_{i}(i=1,2,\cdots ,2n)$:
  (1) $$\zeta_{i}=\frac{\sigma_{i}\frac{\partial G}{\partial x_{i}}|x*}{\sqrt{\sum_{i=1}^{2n}{\left(\sigma_{i}\frac{\partial G}{\partial x_{i}}x*\right)}^{2}}}$$
Step 4: Compute the reliability coefficient *β*:
  (2) $$\beta =\frac{\mu_{z}}{\sigma_{z}}=\frac{G(x_{1}*,x_{2}*,\cdots ,x_{2n}*)+\sum_{i=1}^{2n}(\mu_{i}-x_{i}*)\frac{\partial G}{\partial x_{i}}|x*}{\sqrt{\sum_{i=1}^{2n}{\left(\sigma_{i}\frac{\partial G}{\partial x_{i}}x*\right)}^{2}}}$$
Step 5: Insert *β* into the formula below and obtain the new value of x_i_*:
  (3) $$x_{i}*=\mu_{i}-\beta \zeta_{i}\sigma_{i}$$

Repeat step (2) to step (5) until the difference between the resulting *β* and the previous *β* is less than the tolerance error. At this point the obtained x* = (*x*_1_*, *x*_2_*, …, *x*_2n_*) is the design point to meet the reliability requirements. *x_n_*_+i_* represents the optimum strength value to meet the design requirements and reliability requirements; there is a corresponding x_i_* under each process, establishing a strength interval based on the strength value required by the product.

Stress levels are selected, of which *S*_0_ is the normal stress level, *S_i_* (*i* = 1, 2, …, *n*) is the higher stress level of a kind of stress which is calculated from the stress-strength model. For example, in the case of this paper, three stresses levels were selected: *S*_0_ is the normal level, *S*_1_ is the higher stress level of mechanical stress, and *S*_2_ is the higher stress of thermal stress. The product reliability requirement and design requirement could be met under these kinds of stress:

1. Product failure is caused by only one of the two failure mechanisms, *T*_1_ and *T*_2_ represent the occurrence time of mechanism 1 and mechanism 2, respectively; they are independent of each other, and the product life is *T* = min(*T*_1_, *T*_2_)
2. At the normal stress level, the occurrence time of mechanism *j* (*j* = 1, 2) obeys the Pareto distribution, and its reliability function and inefficiency function are, respectively:
  (4) $$F_{0i}(t)={\theta_{i}}^{\alpha_{i}}t^{-\alpha_{i}},\;t\geq \theta_{i}$$
  (5) $$h_{0i}(t)=\alpha_{i}t^{-1},\;t\geq \theta_{i}$$
  where $\theta_{j}(\theta_{j}>0)$ is the scale parameter, $\alpha_{j}(\alpha_{j}>0)$ is the shape parameter and they are known.
3. At stress level *S_i_*, the failure rate function of mechanism *j* (*j* = 1, 2) is:
  (6) $$h_{ij}(t)=\lambda_{i}\alpha_{j}t^{-1}(\lambda_{i}>1),\;\lambda_{i}\;\mathrm{is}\;\mathrm{the}\;\mathrm{acceleration}\;\mathrm{factor}$$
  (7) $$\bar{F_{ij}}(t)=e^{-\int_{\theta_{j}}^{t}h_{ij}(u)du}={\theta_{j}}^{\lambda_{i}\alpha_{j}}{t^{-}}^{\lambda_{i}\alpha_{j}}$$

The corresponding reliability value is brought into the calculation of acceleration factor $\lambda_{i}$.

## 3. Screening Scheme Evaluation with Defect Stream Analysis

### 3.1. Defect Stream Modeling

**Figure 2:**
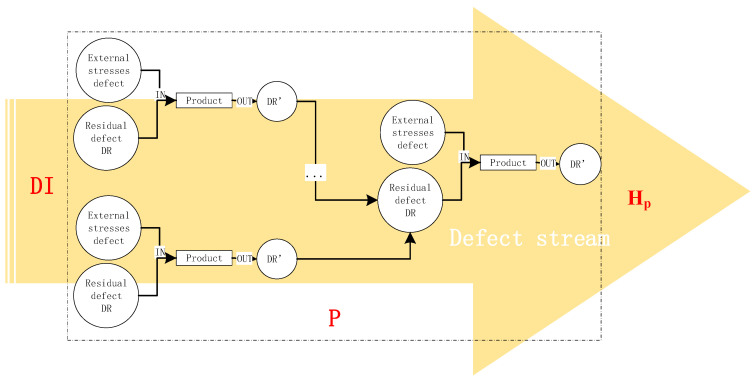
Defect stream of the assembly process.

Different assembly units have different effects on product defects. Set an assembly task as a three-dimensional vector A (DI, P, Hp), where DI is the initial value of defect density, P is the set of processes included in the assembly task, Hp is the value of risk evaluation for this task (Figure 2). The risk of the process can be reduced by the modification of each procedure in the deployment task. Assembly process defect analysis based on defect flow is shown in Figure 2.

This paper also considers the influence of screening stress on product defects to determine the screening scheme. Theoretical analysis shows that after environmental stress screening (ESS), the product will improve the early failure rate. The evaluation of the screening procedure is shown:

(8) $$R(t)=e^{-\lambda_{0}t-D\times [1-e^{-\lambda_{D}t}]}$$

where *λ*_0_ is the specified failure rate determined by the actual target of reliability, *λ_D_* is the early failure rate, *D* is the residual defect density after the ESS, and *T* is the early failure time. *R*_0_(*t*) is the product reliability before screening, *R_a_*(*t*) is the reliability after screening. The change of the evaluation value of the product before and after screening is:

(9) $$R=R_{a}(t)-R_{0}(t)=e^{-\lambda_{0}t-\mu \times D_{R}}-e^{-\lambda_{0}t-\mu \times D_{IN}}$$

### 3.2. Quantification Defines Defect

Various types of defects are introduced in the design and manufacturing process, which are mainly divided into three categories: design defects, technological defects and components defects. However, the design defect cannot be screened by the environmental screen stress. In the actual assembly process, defects are derived from process stress, screening stress and potential defects after the previous procedure. Because the ESS does not screen design defects, if the stress screening is carried out in the previous step, the design defect needs to be subtracted from the current node. This paper assumes that the design defect is a constant value during the assembly process:

(10) $$D_{IN}=D_{Components}+D_{Design}+D_{S}+D_{R}-D_{Design}$$

where $D_{S}$ and $D_{R}$ represent the respective screen defects density and the number of defects density remaining in the previous step. Therefore, the screening degree can be improved indirectly through the analysis of the relationship between various defects of different location nodes.

**Figure 3:**
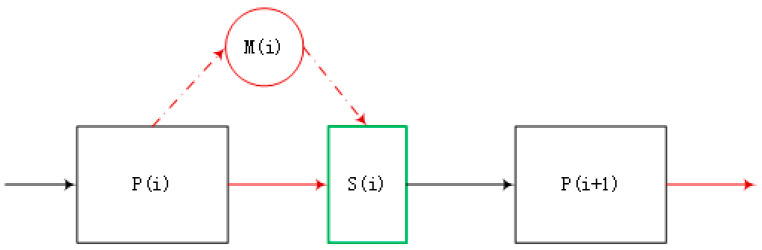
The model of defect density

In Figure 3, the black rectangle represents the process node and the middle (green) square represents the state after screening. If there is no screening, the defect density in the middle (green) square is equal to the density in the black rectangle; in other words, if no new processes are added, the defect density of the product remains unchanged. Conversely, the red one represents the screening node, i.e., the introduced screening stress; the input of the defect would be added. Before the environmental screening stress test, *y*% is the percentage of the defect density of the components. After screening, the ratio of the theoretical value to the actual value of the screening test is *x*%. We establish the relationship between *x* and $y=\frac{D}{D_{IN}},\;x=\frac{F}{M}$, where $D=D_{Process}+D_{Components},\;F=D_{IN}\times TS$, and *TS* represents the degree of screening or indicates the extent to which the process corrects such defects. *M* is the total number of defects after each screening (this article assumes that there are only two types of defects in the assembly process, *M*_1_ is the first type of defect, *M*_2_ is the second type of defect, *M*_1_ + *M*_2_ = *M*). Based on the historical data, the probability density function *D* that shows defect changing process from latent to dominant relative to the time process, was fitted.

It is assumed that the design defect will not be transferred to other defects throughout the process, so *x* after screening is:

(11) $$x=\frac{e^{-\lambda_{0}t-\mu D_{R}}-e^{-\lambda_{0}t-\mu D}}{e^{-\lambda_{0}t-\mu D_{R}}-e^{-\lambda_{0}t-\mu D_{IN}}}=\frac{e^{-\mu D_{R}}-e^{-\mu D}}{e^{-\mu D_{R}}-e^{-\mu D_{IN}}}=\frac{D-D_{R}}{D_{IN}-D_{R}}\times \frac{2-\mu (D_{R}+D)}{2-\mu (D_{R}-D_{IN})}$$

where $\mu =1-e^{-\lambda_{D}t},\;\mu \in [0,1]$ is the parameter according to the specific assembly process. Because of the structure of assembly chain, the method of defect density calculation changes within different location of the nodes. The following two cases are discussed: one case considers the leftmost endpoint node in Figure 2; the other case considers the intermediate nodes. The endpoint node indicates that the product is not elaborated on before this process; for each node, the total number of latent defect depends on the previous procedures. The serial structure and parallel structure would be different for calculating $D_{IN}$. First, the problem is simplified, and the number of left connected nodes in each process is considered to be 1 (*D_ij_* indicates class *i* defect found in operation *j*).

Latent defects and dominant defects exist in the probabilistic assembly process, in which the potential defects are transformed into dominant defects. If the latter is activated, then the product fails, and the next assembly stops. Considering the definition, the potential defects and dominant defects in the assembly process are subject to exponential distribution. The time hypoexponential distribution of the whole assembly process defect change is expressed below.

(12) $$D=f(x)=\frac{\rho_{1}\rho_{2}}{\rho_{1}-\rho_{2}}(e^{-\rho_{2}x}-e^{-\rho_{1}x}),\;x>0$$

In the distribution, two parameters are present ($\rho_{1}\neq \rho_{2}$). The same mean and sample coefficient of variation (*c*) are collected from the history data. The variation coefficient *c* is always < 1. The parameters $\rho_{1}$ and $\rho_{2}$ can be estimated as follows:

(13) $$\rho_{1}=\frac{2}{\bar{x}}{\left[1+\sqrt{1+2(c^{2}-1)}\right]}^{-1}=0.87$$

(14) $$\rho_{2}=\frac{2}{\bar{x}}{\left[1-\sqrt{1+2(c^{2}-1)}\right]}^{-1}=3.2$$

The resulting parameters $\rho_{1}$ and $\rho_{2}$ are real values if $c^{2}\in [0.5,\;1]$. This paper assumes that the total defect is *D*; each time there is a probability $\rho_{1}$ of the first type of defects (component solderability), there is a probability $\rho_{2}$ of the second type of defects (electro migration), where $\rho_{1}$ + $\rho_{2}$ = 1.

### 3.3. Quantitative Characterization of Defect Stream

1. The start node

There is no latent defect introduced from previous operations in the node at the start point:

(15) $$D_{R0}=0,D_{IN}=D+D_{Design}+D_{S}$$

(16) $$x=\frac{D-D_{R}}{D_{IN}-D_{R}}\times \frac{2-\mu (D_{R}+D)}{2-\mu (D_{R}-D_{IN})}=\frac{D-D_{R}}{D_{IN}-D_{R}}=\frac{D\times TS}{M}$$

We simplify this by using power series. μ represents the unreliability of the product, *N* is the number of circle nodes representing the screenings, *TS* is the degree of the screening, and *λ_D_* depends on the stress of the screening. In this paper, *λ_D_* is calculated by the competing failure model based on the stress-strength. The relationship between the degree of ESS and the latent defect (*D_R_* = *D_IN_* (1 − *TS*)) is:

(17) $$Y=\frac{M(1-TS)}{M-TS^{2}},\;D_{R}=\frac{D}{Y}(1-TS)$$

2. The intermediate node

For intermediate nodes representing remaining potential defects after a process, the previous phase is completed in parallel by multiple operations. The reliability of the defects would be greatly reduced because the stress screening needs to eliminate as much as possible the remaining potential defects, reducing the risk of assembly. The failure rate *λ_D_* is calculated by the competing failure model. *D_S_*_1_ represents defects introduced by vibration screening; *D_S2_* represents defects introduced by temperature cycle screening.

(18) $${D_{IN}}_{j}=D_{j}+\sum_{i=1}^{2}(D_{sij}+DR_{i(j-1)})$$

(19) $$y=\frac{D_{j}}{D_{INj}}$$

(20) $$\begin{array}{cc} x & =\frac{D_{j}-\sum_{i=1}^{2}D_{Rij}}{D_{INj}-\sum_{i=1}^{2}D_{Rij}}\times \frac{2-\mu (\sum_{i=1}^{2}D_{Rij}+D_{j})}{2+\mu_{d}\sum_{i=1}^{2}D_{Ri(j-1)}-\mu (\sum_{i=1}^{2}D_{Rij}-D_{INj}+\sum_{i=1}^{2}D_{Ri(j-1)})}\\ & =\frac{D_{j}-\sum_{i=1}^{2}D_{Rij}}{(D_{INj}-\sum_{i=1}^{2}D_{Rij})\mu_{d}\sum_{i=1}^{2}D_{Ri(j-1)}} \end{array}$$

(21) $$D_{INj}=\frac{D_{j}M_{j}}{T{S_{j}}^{2}D_{j}\mu \sum_{i=1}^{2}D_{Rij}-1+M_{j}(1-TS_{j})}$$

It can be concluded that each node is related to the previous node, and the recurrence relationship is as follows:

Only one type of screening is performed at a time when the type of screening is temperature screening (the same applies to the calculation of defect flow in vibration screening):

(22) $$\begin{array}{c} D_{R1j}=\frac{D_{j}p_{1}M_{1j}}{D_{j}p_{1}\mu_{j}\sum_{i=1}^{2}D_{Ri(j-1)}+M_{1j}}\\ D_{R2j}=\frac{D_{j}p_{2}M_{2j}(1-TS_{j})}{T{S_{j}}^{2}D_{j}p_{2}\mu_{j}\sum_{i=1}^{2}D_{Ri(j-1)}+M_{2j}(1-TS_{j})} \end{array}$$

The residual defect density of each node is obtained according to the recursive formula. To generalize the problem, the left connected node is not 1. This means that there is no parallel assembly of multiple operations before a single operation. In other words, there are more processes that affect the defect density of the product. The calculation of parallel nodes mainly considers the residual defect density in the combined node, as shown below: in this paper, m is the number of the left nodes:

(23) $$D_{INj}=D_{j}+\sum_{j}^{m}\sum_{i=1}^{2}D_{Ri(j-1)}D_{Si}$$

The parallel system is assembled from multiple parts to components, so the stress levels in each part are different, and the values of each residual defect density are different. The residual defect density after screening is as follows: only one type of screening is performed at a time, when the type of screening is temperature screening (the same applies to the calculation of defect flow in vibration screening):

(24) $$\begin{array}{c} D_{R1j}=\frac{D_{j}p_{1}M_{1j}}{D_{j}p_{1}\mu_{j}\sum_{j}^{m}\sum_{i=1}^{2}D_{Ri(j-1)}+M_{1j}}\\ D_{R2j}=\frac{D_{j}p_{2}M_{2j}(1-TS_{j})}{T{S_{j}}^{2}D_{j}p_{2}\mu_{j}\sum_{j}^{m}\sum_{i=1}^{2}D_{Ri(j-1)}+M_{2j}(1-TS_{j})} \end{array}$$

For series and parallel nodes, the residual defect density of the current node can be represented by a recursive equation. The other parameters can be analyzed by the specific assembly process.

### 3.4. Screening Scheme Evaluation

The total number of defects $D_{IN}$ in the product after the assembly process is the input *I*; the defective products are detected by the buffer after the process; they return to the buffer before the process if this process needs to be screened. The residual potential defects $D_{R}$ in the final output of the assembly process after *n* tests are *O*; no screening, *O* = *I*. When the input is greater than the output, the assembly process risk is low and can meet the requirements of the assembly; when the input is less than the output, the high risk of the assembly process cannot meet the requirements. The purpose of the study of assembly process risk is to reduce the assembly process risk and improve the assembly quality by optimizing the assembly process and increasing the screenings.

The process risk of the assembly process is evaluated by the entropy. Entropy represents the degree of confusion within the system. Accordingly, if the entropy is close to 1, the system is more chaotic, and the control ability of the system is poor. The calculation of entropy not only characterizes the risk of assembly process but also characterizes the complexity of the assembly process, which can help us make better decisions. This means the entropy of each process *H_pi_* and the entropy of the assembly process *H* are calculated in this way:

(25) $$H_{pk}=-\frac{O_{k}}{I_{k}}\log_{2}\frac{O_{k}}{I_{k}},\;H=-\frac{O_{n}}{I_{0}}\log_{2}\frac{O_{n}}{I_{0}}$$

where *k* = [0, *n*], *k* ∈ *N*, *n* is the total nodes of the assembly process. Based on the residual defect analysis, the evaluation of the screening procedure is calculated as the change of the residual defect density:

(26) $$R(t)=e^{-\lambda_{0}t-D_{R}\times [1-\exp(-\lambda_{D}t)]}$$

where *t* is the total time required to complete the current process. Combined with the previous analysis, we obtain:

(27) $$I=e^{-\lambda_{0}t-D_{IN}\times [1-\exp(-\lambda_{D}t)]}\;O=e^{-\lambda_{0}t-D_{R}\times [1-\exp(-\lambda_{D}t)]}$$

We find the best screening method of the assembly process by means of recursive optimization. The derivation process of the recursive relation will be explained in detail later.

## 4. Case Study

**Figure 4:**
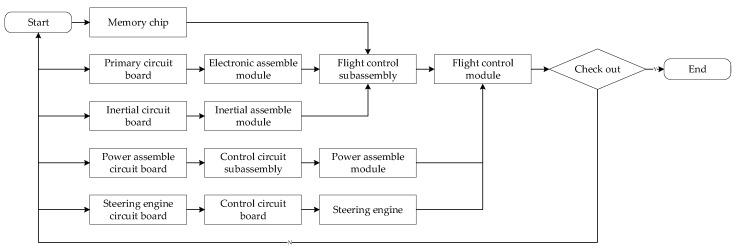
The flow chart of the assembly process of a flight control module.

At present, PCBAs play an increasingly important role in various fields and have become more popular and customized. In the manufacturing process of PCBAs, the workload of the assembly process accounts for more than 50% of the total workload. The assembly process of the flight control of a PCBA is analyzed as an example. As a fragile part in the assembly process, PCB is analyzed in this case mainly for the defects of the PCB board in the assembly process. Using work breakdown structure (WBS) to find the key components, the flow chart of these key components in the assembly process is shown in Figure 4.

Steps 1–4 describe the process of using the method in this paper for this case: 

Step 1: Determine the process flow and the number of the process

**Figure 5:**
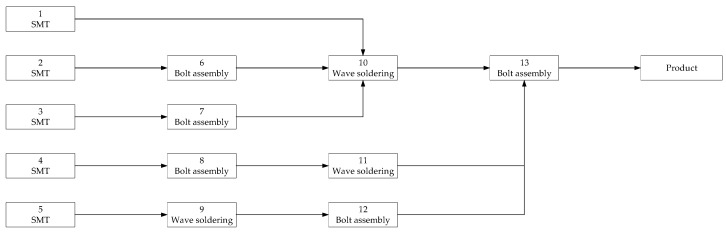
The flow-chart of the process.

The main processes of the assembly process are: mechanical clamping, surface mount technology (SMT) and wave soldering. All the processes are shown in Figure 5.

Step 2: Fitting the function of stresses

Monitor the previous process by the sensor and collect the stress data of the assembly process to fit the stress distribution. Select the key position on the assembled workpiece. 

The optimized function *F*(*x*) of loading stress with 95% confidence bounds is:

(28) $$F(x)=-0.0012x^{7}-0.000274x^{5}+0.00058x^{4}+0.0000683x^{3}+0.0049x^{2}-0.18x+2.907$$

The optimized function *F*(*x*) of the SMT process with 95% confidence bounds is:

(29) $$\begin{array}{cc} F(x)= & 279\times \sin(0.00915x-0.1247)+110.2\times \sin(0.0016x+2.043)+\\ & 12.06\times \sin(0.054x-0.6275)+14.49\times \sin(0.0436x-2.461)+\\ & 7.633\times \sin(0.098x-9.06)+6.973\times \sin(0.098x-0.363) \end{array}$$

The optimized function *F*(*x*) of the waving welding process with 95% confidence bounds is:

(30) $$\begin{array}{cc} F(x)= & 91.09\times e^{-{\left(\frac{\left(x-142.7\right)}{3.004}\right)}^{2}}+100.1\times e^{-{\left(\frac{\left(x-139.1\right)}{0.9736}\right)}^{2}}+55.67\times e^{-{\left(\frac{\left(x-147.2\right)}{4.666}\right)}^{2}}-\\ & 180.7\times e^{-{\left(\frac{\left(x-160.5\right)}{8.101}\right)}^{2}}+248.4\times e^{-{\left(\frac{\left(x-159.9\right)}{9.4}\right)}^{2}}+44.75\times e^{-{\left(\frac{\left(x-127.7\right)}{17.67}\right)}^{2}}+\\ & 2592\times e^{-{\left(\frac{\left(x-1301\right)}{624}\right)}^{2}}+38.36\times e^{-{\left(\frac{\left(x-97.45\right)}{25.53}\right)}^{2}} \end{array}$$

**Figure 6:**
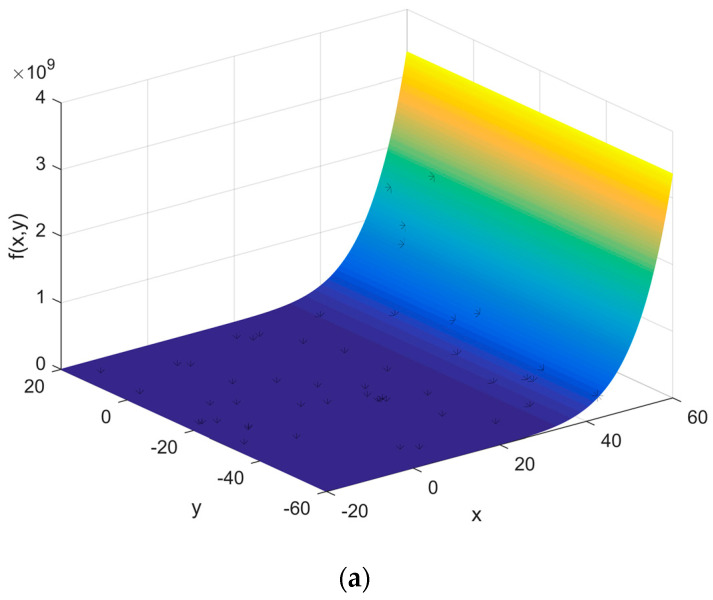

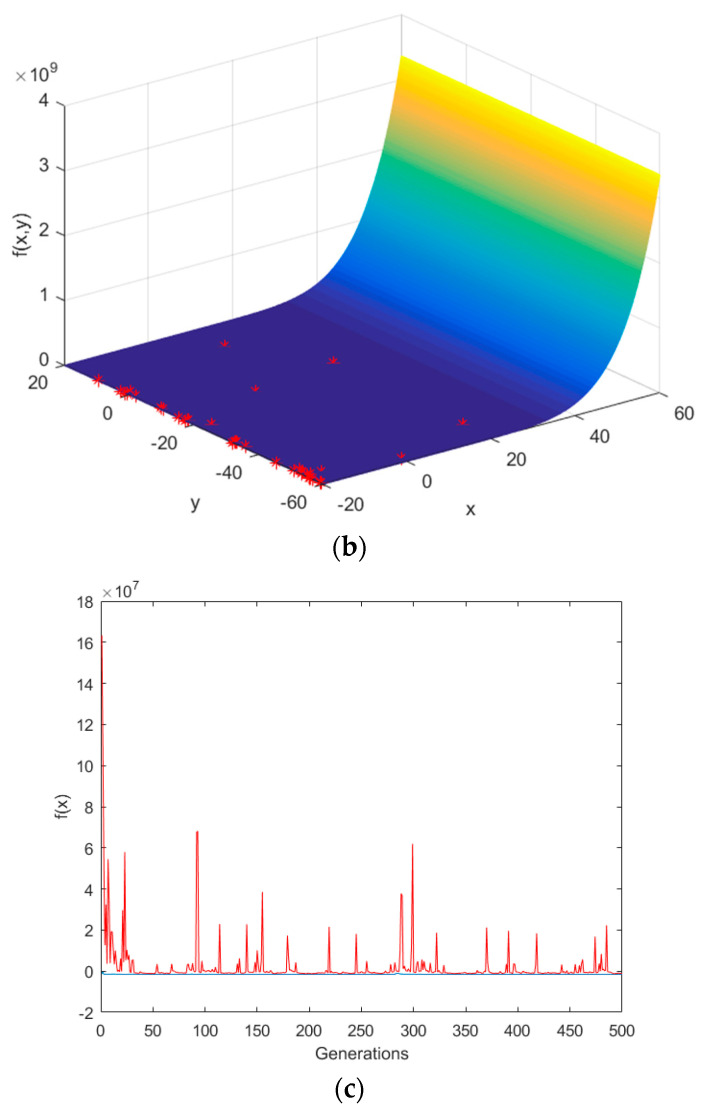
Maximum loading stress found. (**a**) The initial position of the chromosome; (**b**) The final position of the chromosome; (**c**) The optimization process.

**Figure 7:**
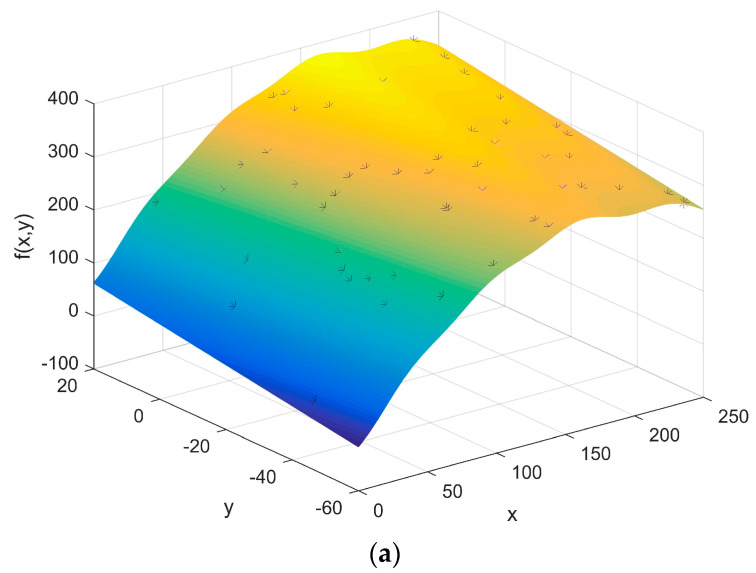

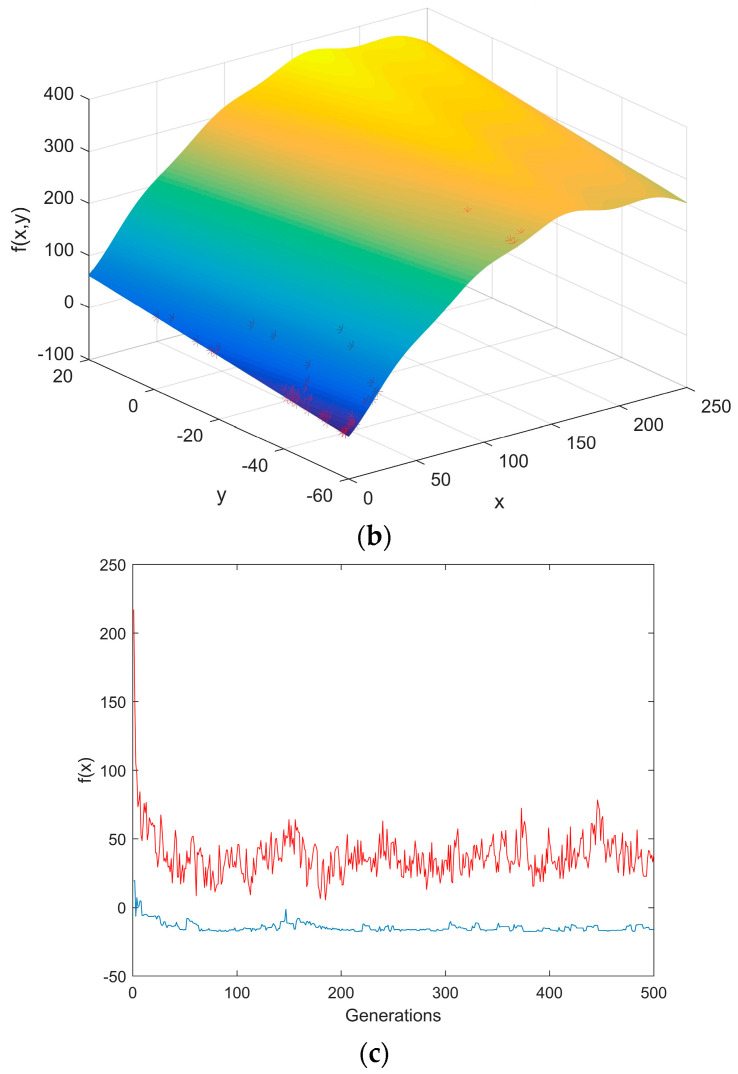
Maximum SMT found. (**a**) The initial position of the chromosome; (**b**) The final position of the chromosome; (**c**) The optimization process.

**Figure 8:**
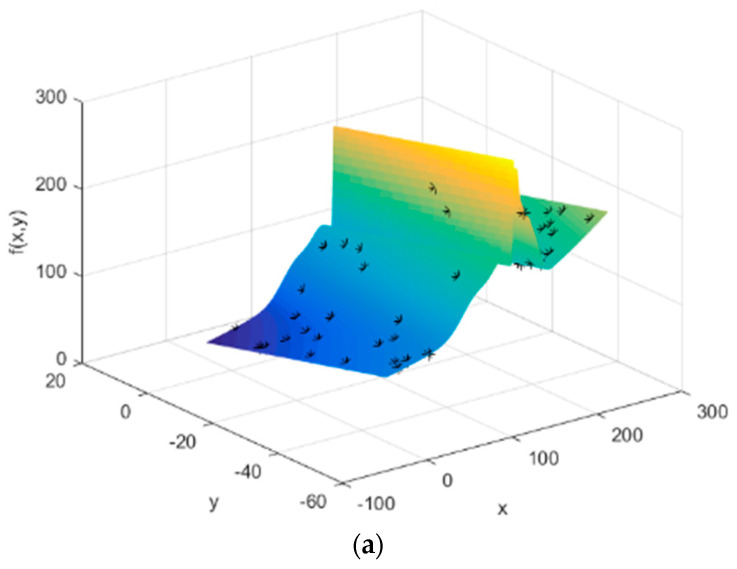

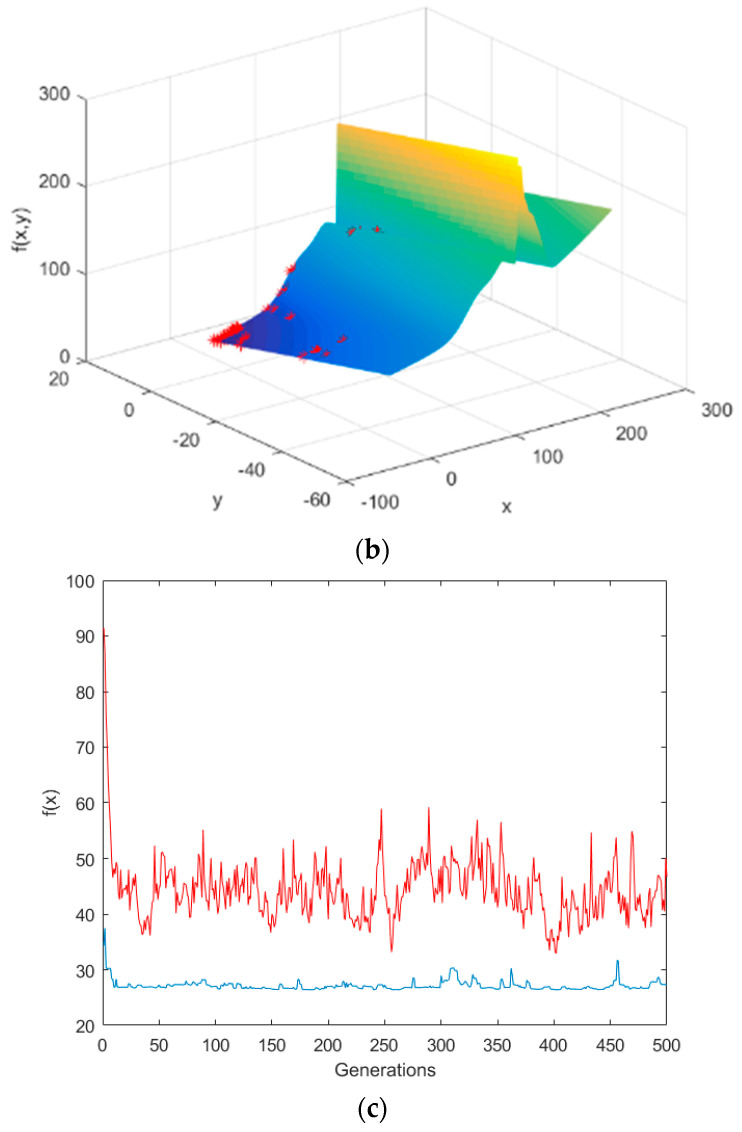
Maximum waving welding found. (**a**) The initial position of the chromosome; (**b**) The final position of the chromosome; (**c**) The optimization process.

The stress distribution is brought into the multivariate function *Z* and the minimum value of *Z* in the range of stress and strength is obtained by genetic algorithm. At this point, the optimal stress value for the maximum strength under the guaranteed reliability level of the process is calculated. The calculation process is shown in Figure 6, Figure 7 and Figure 8 and the results are shown in Table 1.

Step 3: As far as the above three stresses are concerned, the effect of the mechanical assembly process on product quality should be paid more attention in the assembly process.

Three stress levels are selected, of which *S*_0_ is normal stress level, *S*_1_ and *S*_2_ are the high levels of loading stress and hot stress, respectively; the product reliability requirement and design requirement could be met under three kinds of stress:

1: Product failure is caused by only one of the two failure mechanisms; *T*_1_ and *T*_2_ represent the occurrence time of mechanism 1 and mechanism 2, respectively, and they are independent of each other; the product life is *T* = min(*T*_1_, *T*_2_).

2: At the normal stress level, the occurrence time of mechanism *j* (*j* = 1, 2) obeys the Pareto distribution, and its reliability function and inefficiency function are, respectively:

(31) $$F_{0j}(t)={\theta_{j}}^{\alpha_{j}}t^{-\alpha_{j}},\;t\geq \theta_{j}$$

(32) $$h_{0j}(t)=\alpha_{j}t^{-1},\;t\geq \theta_{j}$$

where $\theta_{j}(\theta_{j}>0)$ is the scale parameter, $\alpha_{j}(\alpha_{j}>0)$ is the shape parameter and they are known.

3: At stress level S_1_, the failure rate function of mechanism *j* (*j* = 1, 2) is:

(33) $$h_{1j}(t)=\lambda_{1}\alpha_{j}t^{-1},\;h_{2j}(t)=\lambda_{2}\alpha_{j}t^{-1}(\lambda_{1},\;\lambda_{2}>1),\;\lambda_{1},\;\lambda_{2}\;\mathrm{is}\;\mathrm{the}\;\mathrm{acceleration}\;\mathrm{factor}$$

(34) $$\bar{F_{1j}}(t)=\exp(-\int_{\theta_{j}}^{t}h_{1j}(u)du)={\theta_{j}}^{\lambda \alpha_{j}}{t^{-}}^{\lambda \alpha_{j}},\;\bar{F_{2j}}(t)={\theta_{j}}^{\lambda \alpha_{j}}{t^{-}}^{\lambda \alpha_{j}}$$

The corresponding reliability value is brought into the calculation of acceleration factor λ. $\alpha_{1}=0.2,\alpha_{2}=0.3$; the result of the parameter fitting is shown in Table 2.

Obtain the acceleration factor to calculate the failure rate:

(35) $$h_{1j}(t)=1.179\times \alpha_{j}t^{-1},\;h_{2j}(t)=1.867\times \alpha_{j}t^{-1}$$

Step 4: Screening optimization

**Figure 9:**
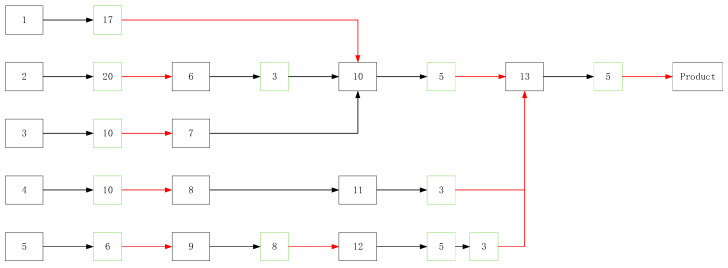
Historical screening program.

The assembly chain is numbered, and the relevant parameters are calculated according to the original assembly sequence and selection scheme. Figure 9 is an existing assembly scheme. We analyze and optimize this process.

In Figure 9 the green square is the node of screening, and the number in the green square represents the temperature cycles (where, for this case, the range of the temperature cycle is −50 °C–70 °C (*R* = 120), the rate of the temperature change v is 10), and the red connector indicates 20 min of random vibration filtering. For this screening program, after the process of No. 7 and No. 8 without screening, No. 9 conducted a second screening. The number of problems in the actual production process, the screening strength and the screening position of each screening point of the original screening scheme, and the failure rate of the assembly process, are obtained through the historical production data. Combined with the recursive function of residual defect density DR deduced in this paper, we calculate the residual defect density in every node of this assembly process by the original screening scheme. The other parameters are calculated before with the historical assembly process data. No. 10 and No. 11 are parallel nodes, and the remaining nodes are series nodes.

(36) $$TS_{h}=1-e^{-0.0017\times {\left(R+0.6\right)}^{0.6}\times [\ln{\left(e+v\right)}^{3}]\times N},\;TS_{l}=1-e^{-0.0046(Grms)\times 1.71\times t}$$

**Figure 10:**
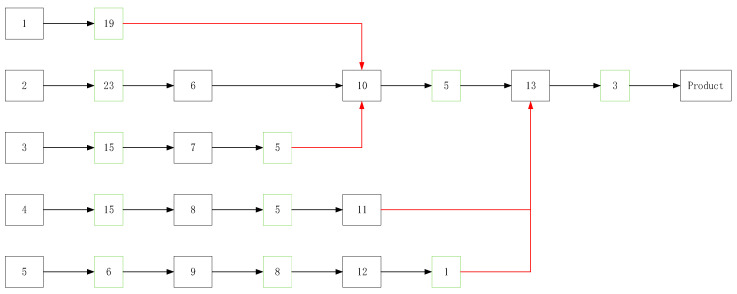
Screening optimization.

As shown in Table 3, the first point, after the secondary screening of the node No. 12, is that the defect of the screening stress increases the intrinsic residual defect density in the product. The entropy of the low-levels, the nodes in which *I* = 1, is less than in the other parts. However, under the good screening scheme, the number of problems should decrease with the increase of *j*. So, we need to strengthen the low-level screening. Based on the recursive relationship of defect density, the optimal selection scheme is found and is shown in Figure 10.

Based on this program, the entropy of each node is in Table 4.

## 5. Summary and Conclusions

In this study, the multistage assembly process is analyzed, and the effect of defect changes on product quality during the assembly process is also investigated. Through the analysis of the assembly task, the recursive relationships of residual defect density under multi-stress are obtained, and the defect analysis is quantified. The results of the probability density function that shows the defect changing process from latent to dominant upon the time process agree with the historical data. The definition of entropy in physics was used to characterize the risk of the model that evaluates the assembly process and the screening effects. Finally, we analyze a case for a flight control module assembly process in a PCBA to illustrate the effectiveness of our approach. Our result shows that the screening program can be improved.

In this paper, the effect of multiple stresses on different kinds of defects is not fully characterized quantitatively. Therefore, we will focus on the coupling effect of multi-stress on product quality for more precise selection of defects produced in the assembly process in our future work.

## Figures and Tables

**Table 1:** The result of the stress-strength model.

Process	Stress	Strength	Reliability
Loading	4.9982	3.4424	0.6331
SMT	139.1388	5.1437	0.7967
Waving Welding	163.8467	6.1099	0.8315

**Table 2:** Parameter fitting result.

Parameters	MLE	Stud-t	HPD
$\lambda_{1}$	1.179	(0,2.497)	(1.500,2.005)
$\lambda_{2}$	1.867	(0.3,2.109)	(1.36,1.938)
$\theta_{1}$	2.405	(2.235,2.591)	(2.099,2.423)
$\theta_{2}$	3.347	(2.446,4.538)	(2.930,3.676)

**Table 3:** The entropy of the assembly process.

No.	SS	Number of Problems	*D_IN_*	*D_R_*	*H_p_*
1	99.978%	20	200	10.52607	0.389368
2	99.995%	83	350	4.268288	0.37276
3	99.297%	37	200	5.553395	0.38342
4	99.297%	65	400	6.248633	0.37886
5	94.894%	32	183	5.884339	0.41831
6	77.405%	8	7.665	1.544	0.61974
7		\	44.34	49.89395	1
8		\	69.2	75.448633	1
9	98.106%	28	6.105	2.3578	0.55391
10	91.618%	27	63.30295	19.247	0.55757
11	77.405%	15	74.014	8.1467	0.58672
12	91.618%;77.405%	12;1	49.2298	15.91;37.55	0.85931
13	99.941%	16	78.596	20.319	0.47683
product					0.37322

**Table 4:** The entropy of the new screening.

Number	SS	$H_{p}$	Number of Problems
1	0.999919	0.270838	23
2	0.999989	0.285324	135
3	0.999411	0.306212	67
4	0.999411	0.051508	65
5	0.999411	0.164528	48
6	0	1	\
7	0.916178	0.167138	32
8	0.916178	0.09126	28
9	0.98106	0.026507	28
10	0.916178	0.057906	13
11	0	1	\
12	0.390924	0.09801	8
13	0.774049	0.026507	3
Product		0.0313	
